# The exported chaperone Hsp70-x supports virulence functions for *Plasmodium falciparum* blood stage parasites

**DOI:** 10.1371/journal.pone.0181656

**Published:** 2017-07-21

**Authors:** Sarah C. Charnaud, Matthew W. A. Dixon, Catherine Q. Nie, Lia Chappell, Paul R. Sanders, Thomas Nebl, Eric Hanssen, Matthew Berriman, Jo-Anne Chan, Adam J. Blanch, James G. Beeson, Julian C. Rayner, Jude M. Przyborski, Leann Tilley, Brendan S. Crabb, Paul R. Gilson

**Affiliations:** 1 Burnet Institute, Melbourne, Victoria, Australia; 2 Department of Biochemistry and Molecular Biology, The University of Melbourne, Melbourne, Victoria, Australia; 3 Bio21 Molecular Science and Biotechnology Institute, The University of Melbourne, Melbourne, Victoria, Australia; 4 Wellcome Trust Sanger Institute, Wellcome Genome Campus, Hinxton, Cambridgeshire, United Kingdom; 5 Walter & Eliza Hall Institute, Melbourne, Victoria, Australia; 6 Parasitology, FB Biology, Philips University Marburg, Marburg, Germany; 7 Monash University, Melbourne, Victoria, Australia; Bernhard Nocht Institute for Tropical Medicine, GERMANY

## Abstract

Malaria is caused by five different *Plasmodium* spp. in humans each of which modifies the host erythrocyte to survive and replicate. The two main causes of malaria, *P*. *falciparum* and *P*. *vivax*, differ in their ability to cause severe disease, mainly due to differences in the cytoadhesion of infected erythrocytes (IE) in the microvasculature. Cytoadhesion of *P*. *falciparum* in the brain leads to a large number of deaths each year and is a consequence of exported parasite proteins, some of which modify the erythrocyte cytoskeleton while others such as *Pf*EMP1 project onto the erythrocyte surface where they bind to endothelial cells. Here we investigate the effects of knocking out an exported Hsp70-type chaperone termed Hsp70-x that is present in *P*. *falciparum* but not *P*. *vivax*. Although the growth of Δ*hsp70-x* parasites was unaffected, the export of *Pf*EMP1 cytoadherence proteins was delayed and *Δhsp70-x* IE had reduced adhesion. The Δ*hsp70-x* IE were also more rigid than wild-type controls indicating changes in the way the parasites modified their host erythrocyte. To investigate the cause of this, transcriptional and translational changes in exported and chaperone proteins were monitored and some changes were observed. We propose that *Pf*Hsp70-x is not essential for survival *in vitro*, but may be required for the efficient export and functioning of some *P*. *falciparum* exported proteins.

## Introduction

Nearly half of the world’s population (3.2 of 7.1 billion) are at risk from contracting malaria, caused by the *Plasmodium* genus of intracellular parasites, which can cause severe life threatening disease and debilitating illness [1]. *Plasmodium* parasites amplify in the erythrocytes of their host animals, and to do so the parasites must remodel their host cells in order to import plasma nutrients and evade the immune system. The most pathogenic *Plasmodium* species to humans is *Plasmodium falciparum (Pf)*, which can cause severe morbidity and mortality. Many of the severe consequences in *Pf* infections are due to expression of an erythrocyte surface exposed exported protein, erythrocyte membrane protein 1 (*Pf*EMP1). In a process known as cytoadherence, *Pf*EMP1 allows the infected erythrocytes (IE) to bind endothelial cells and avoid splenic clearance [2]. Cytoadherence can however occlude the microvasculature and cause severe pathogenesis, which in the brain can result in coma and death, and in the placenta of pregnant women can lead to fetal growth restriction and low birth weight, or pre-term birth [2–5].

In order to remodel their host cells *Pf* parasites export effector proteins into the erythrocyte cytoplasm and plasma membrane [5, 6]. These proteins must first traverse the parasite’s plasma membrane into the parasitophorous vacuole (PV) space and then across the enveloping PV membrane (PVM). Proteins cross the PVM in an unfolded state [7] via a proteinaceous pore termed PTEX [8]. Most proteins destined for export via PTEX contain a pentameric PEXEL motif, although there are also many PEXEL-negative exported proteins (PNEPs) [8–12]. There are five core components of PTEX including HSP101, a Hsp100 chaperone that when functionally ablated causes rapid parasite death [13]. Hsp100 chaperones typically interact with other chaperone components, including Hsp70/Hsp40 pairs that can unfold and refold complex proteins [14, 15]. We recently identified a novel Hsp70, Hsp70-x, that is secreted into the PV where it can interact with PTEX, as well as being exported into the host erythrocyte [16–18].

Hsp70-x is specific to the *Laverania* sub-genus of *Plasmodium*, which includes *P*. *falciparum* and multiple related species that infect great apes [18]. Hsp70-x is absent from other *Plasmodium* species including *P*. *vivax*, the other major cause of human malaria. Functions common to *Laverania* species are the ability to invade both mature and immature erythrocytes known as reticulocytes [19], a large exportome of hundreds of PEXEL proteins [20], and an ability to strongly cytoadhere via surface virulence factors such as *Pf*EMP1 [21, 22]. In contrast, *P*. *vivax* only invades reticulocytes, exports fewer PEXEL proteins, and does not cytoadhere to the same extent resulting in reduced virulence [23–25]. We hypothesized that the presence of Hsp70-x in *P*. *falciparum* and *P*. *reichenowi* could be a contributing factor to one or all of these *Laverania* specific features.

Here we report on the successful genetic deletion of *hsp70-x* in *P*. *falciparum* resulting in no effect on growth or stress response *in vitro*. The ΔHsp70-x parasites however have reduced virulence characteristics in *in vitro* assays, and the IE are less cytoadherent under flow conditions and are more rigid which might reduce their fitness *in vivo*. The parasites could have responded to genetic ablation of Hsp70-x by increasing the transcription and translation of some chaperones and exported proteins.

## Materials and methods

### Plasmid construction

To make a functional knockout of *hsp70-x* two flanking regions of ~1kb (Flank 1 and Flank 2) were cloned from the genomic DNA of the *P*. *falciparum* strain, 3D7. Flank 1 was amplified using primers TACATACCGCGGGTAACAATGCAGAAGAATCAGAGGTTGCA and TTTCAACTAGTATTAAAGTATTACGGAATTGATCCAT, and Flank 2 using primers CCAAGTCCATGGAATTGTTTTAGTTGGTGGTTCA and CTTTATCCTAGGAATTACGACCTCTTAAGTTGGTTGCCT, with restriction sites underlined. The flanks were cloned into plasmid pCC1 either side of the DHFR cassette and double homologous recombination was driven by cytosine deaminase. Knockout was confirmed by PCR with primer pairs A/A’ for *hsp70x* GCAAGAAACAACCTAGAAAATTACTG and GGAAACAATCCAAATGTTGTTGC, B/B’ for cytosine deaminase ACACAGTAGTATCTGTCACCAAAGTCA and GTGACAGGGGGAATGGCAAGC, C/C’ for *hsp70-x* 5’UTR TGCAACAACAACAGTACATACAGCAAGT and *hDHFR* ACGATGCAGTTTAGCGAACCATGC and D/D’ for *hDHFR* CTGATGTCCAGGAGGAGAAAGGCAT and *hsp70x* 3’UTR TGGTTTAATTTACTTCTTCAACGGTTGGTCCA.

### *P*. *falciparum* culture and transfection

Parasites were grown as per a modified version of Trager and Jenson [26]. Briefly, parasites were grown in complete RPMI-1640-HEPES media (Sigma) supplemented with 0.25% AlbumaxII (Gibco) and 5% heat inactivated pooled human sera (Australian Red Cross Blood Service). Red blood cells (Australian Red Cross Blood Service) were transfected with 150 μg of plasmid DNA and trophozoites added. Single homologous recombination was driven with 6 cycles with 2.5nM WR99210. Parasites were successfully recovered and subjected to negative selection with 5-fluorocytosine [27] on alternate days for 5 days followed by continuous culture. Recovered parasites were cloned by limiting dilution.

### Growth assays

Malstat assay to measure lactate dehydrogenase (LDH) correlates with parasitaemia and was performed as described previously [28]. Parasitaemia was adjusted to 0.2% parasitaemia in 2% haematocrit and seeded in triplicate and samples stored at -80°C. Assays were performed at the trophozoite stage in the absence of drug selection as described previously and absorbance measured at 630nm on Multiskan GO (Thermo Scientific).

### Flow cytometry

iRBC at 0.04% haematocrit were washed once in 0.1% casein in PBS, and centrifuged at 2000g for two min. Cells were resuspended in 10 μg/mL ethidium bromide (Invitrogen) in 0.1% casein in PBS and incubated in the dark at room temperature for 20–40 minutes. Cells were washed and resuspended in PBS. Samples were analysed on a FACSCanto II. Labelling of IEs by antibodies present in serum from malaria-exposed pregnant women, to evaluate *Pf*EMP1 surface expression was performed as described previously [12]. Serum samples were obtained from pregnant women resident in a malaria-endemic region of Papua New Guinea and were previously tested to identify samples with antibody reactivity to the surface of CS2 IEs [29, 30]. Ethics approval was obtained from the Medical Research Advisory Committee, PNG, and the Human Research and Ethics Committee of Alfred Health, Australia. All subjects gave written informed consent.

### Immunofluorescence assay (IFA)

IFAs were performed as described previously [12]. Briefly, the IFAs were performed on dried blood smears fixed for 5 minutes with cold 100% methanol. After drying the slides were blocked in 3% BSA. Primary antibodies were added at 1:500 of serum in 3% BSA. Secondary antibodies (AlexaFluor 488, 568 or 594) were added at 1:2000. Imaging was performed on a Zeiss Axio Observer ZV1 and analysed with Fiji v1.4. Measurement of mean fluorescence intensity was performed using Fiji by manually selecting the area outside the PVM marker EXP2, and within the erythrocyte boundary by DIC/Bright field.

### Western blot analysis

Mixed ring and trophozoite stage parasites were passed through a magnetized column (Miltenyi) to separate trophozoites from rings. Whole trophozoite infected erythrocytes were resuspended in about 30 pellet volumes of SDS sample buffer and fractionated on 4–12% gels (Life Technologies) by SDS PAGE. The ring samples were lysed in 0.09% saponin, and washed in PBS to remove haemoglobin. The ring pellet was similarly prepared for SDS-PAGE. After transfer to nitrocellulose membrane the blots were probed with rabbit serum for KAHRP and SBP1 (kind gifts from Alan Cowman and Brian Cooke) at 1/500 in 1% casein. Rabbit serum for Hsp70-1 was produced by the Walter & Eliza Hall Institute Monclonal facility and was similarly used at 1/1000. Mouse monoclonals for RESA 18.2 (a kind gift from Robin Anders) and EXP2 were used at 3 μg/mL. Secondary antibodies labeled with 700 and 800 nm fluorescent dyes were used at 1/5000 and detected with an Odyssey FC scanner (Li-Cor). Densitometry was performed using the Odyssey Image Studio software.

### Cytoadhesion and cell rigidity assays

Cytoadhesion under flow conditions was performed as described previously [31], using a flow chamber coated with chondroitin sulfate A (CSA) at 100 μg/mL in PBS and imaged on a DeltaVision microscope. Static flow was analysed with CSA bound to plastic dishes at 40 μg/mL. Cell deformability using artificial spleen model was performed as described previously [32]. Cells were synchronized by heparin and sorbitol to within a four hour window.

### Scanning electron microscopy (SEM)

Cells were synchronized by heparin and sorbitol, then magnet purified to >90% iRBC at trophozoite stage and fixed in PBS with 1% glutaraldehyde, and stored at 4°C until needed. IE were washed in PBS with 3 centrifugation cycles (2 min at 300*g*) before being deposited onto coverslips coated with poly-L-lysine. Cells were dehydrated in a series of ethanol and water baths at 0, 20, 50, 70, 80, 90, 95, and 100% (x3) ethanol for 5 min each. Coverslips were coated with approximately 2 nm of Au prior to imaging in a FEI Teneo microscope operating at 2 kV with backscatter detection, using a working distance of 5 mm.

### RNA-seq

Parasites were synchronized with sorbitol and heparin, and total RNA was prepared from different staged samples as per [33]. A Bioanalyzer Nano chip (Agilent) was used to QC and quantify total RNA. A modified RNA-seq protocol (“DAFT-seq”, [34]) was used to account for the extreme AT-content of the *P*. *falciparum* transcriptome. Briefly, polyA+ RNA (mRNA) was selected using magnetic oligo-d(T) beads (E7490, NEB). Reverse transcription using Superscript II (Life) was primed using oligo d(T) primers, then second strand cDNA synthesis included dUTP. The resulting cDNA was fragmented using a Covaris AFA sonicator (20% duty cycle; intensity 5; 200 cycles per burst for 30 s). A “with-bead” protocol was used for dA-tailing, end repair and adapter ligation (NEB) using “PCR-free” barcoded sequencing adaptors (similar to [35]). After 2 rounds of SPRI cleanup the libraries were eluted in EB buffer and USER enzyme mix (NEB) was used to digest the second strand cDNA, generating directional libraries. Samples were sent for sequencing without amplification where possible. For lower input samples (10–12 hours post invasion (hpi) and 20–24 hpi) libraries were amplified by 16 cycles of PCR with KAPA HIFI polymerase [36]. The libraries were quantified by qPCR and sequenced on an Illumina HiSeq2000. Data was analysed at the Wellcome Trust Sanger Institute, UK. Reference *Plasmodium* genomes for 3D7 and IT were downloaded from GeneDB [37]. Reads were mapped with TopHat [38]. Differential expression was analysed using DESeq [39]. RNA-seq data was visualized in Artemis [40].

### Stable-isotope labeling and chromatography mass spectrometry detection (SILAC)

SILAC labelling of blood-stage parasites was performed as previously outlined [41]. Briefly, parasites were grown for three cycles in heavy or light isoleucine media, and synchronized with sorbitol. Age-matched 20–28 hpi trophozoites were magnet purified and mixed in equal ratios. Equinatoxin in PBS was used to lyse the RBC membrane [42] and supernatant was collected. Haemoglobin was removed using Ni+ NTA resin (Sigma) prior to mass spectrometry performed exactly as described previously [16].

## Results

### Hsp70-x is not essential to survival

**Fig 1:**
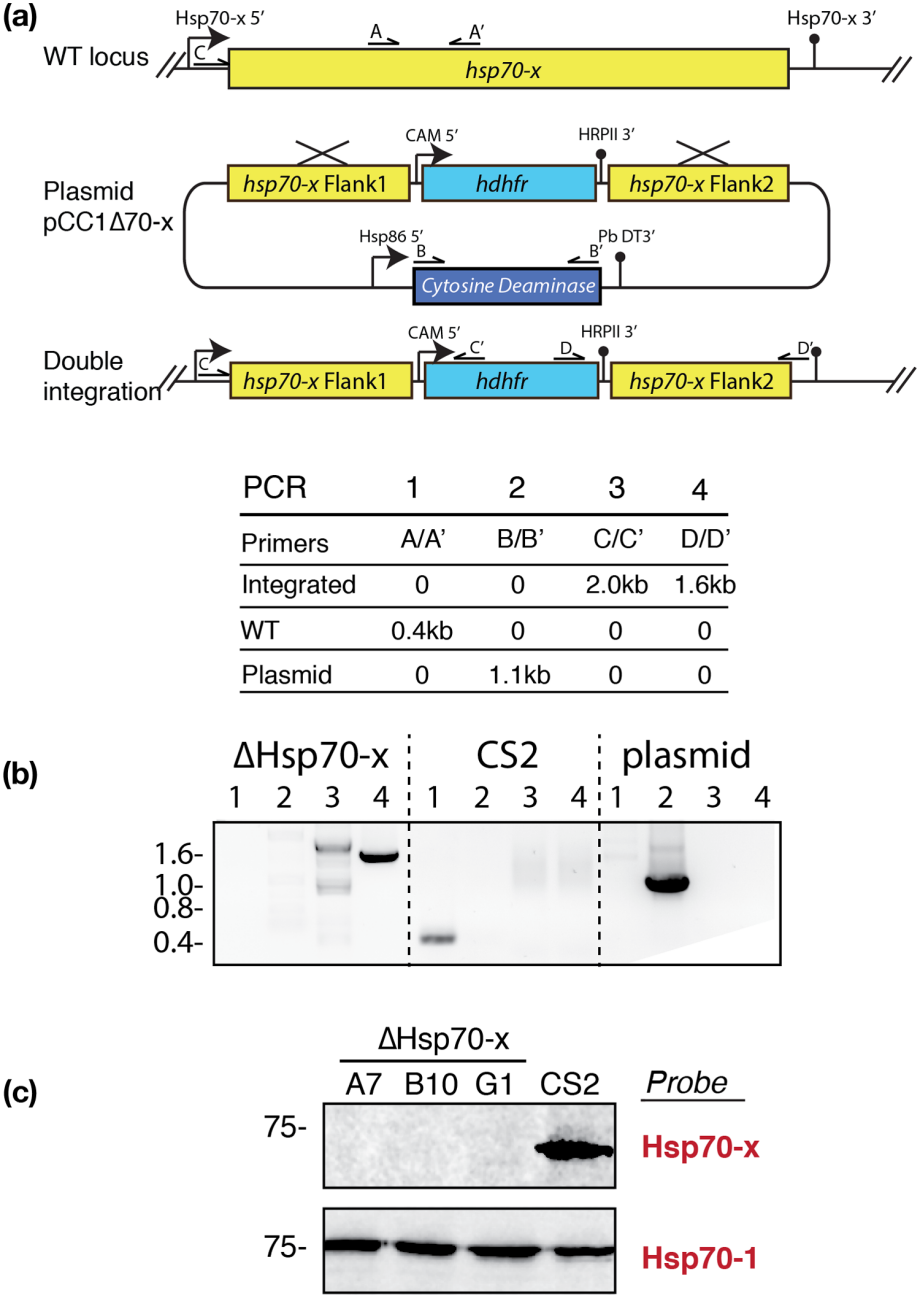
*hsp70-x* can be knocked out and is not essential for parasite survival. (a) Diagram of plasmid construct indicating double homologous recombination event to replace *hsp70-x* with a *hdhfr* drug resistance cassette. Negative selection pressure to remove parasites carrying non-integrated plasmid is provided by the cytosine deaminase. The binding sites of PCR primers used to confirm gene replacement are indicated. (b) (Top) Table of PCR primer combinations and expected DNA product sizes resulting from amplification the native *hsp70-x* locus, the *hdhfr* replacement locus and plasmid construct are shown. (Bottom) Agarose gel of PCR products from one Δ*hsp70-x* clone, the parental (CS2) parasites and plasmid only controls indicate the *hsp70-x* gene has been deleted by double cross over integration. (c) Western blot probed with rabbit anti-Hsp70-x confirms three separate ΔHsp70-x clones no longer express Hsp70-x, but retain the closely related cytoplasmic Hsp70-1.

The *P*. *falciparum* line CS2 was used as the parental line for *hsp70-x* deletion as it expresses the stable and well characterized *Pf*EMP1 variant, VAR2CSA [5, 43]. To determine whether *P*. *falciparum* requires Hsp70-x to survive in mature erythrocytes or to export and functionalize its large exportome we attempted to delete the *hsp70-x* gene. A double homologous recombination method was used to replace *hsp70-x* with a human dihydrofolate reductase gene (*hdhfr*) cassette that conferred resistance to the antifolate WR99210 (Fig 1a). After multiple attempts, viable parasites were eventually recovered after six cycles of selection with WR99210 and negative selection cycles with 5-fluorocytosine to remove non-integrated plasmids. Anecdotally this indicated to us that deletion of *hsp70-x* was difficult to achieve.

Three clonal ΔHsp70-x lines (A7, B10, G1) were established by limiting dilution, and the double recombination and gene replacement was confirmed by PCR (Fig 1b). To verify that *hsp70-x* had been ablated, western blot analysis was performed using antibodies specific to Hsp70-x (Fig 1c) [18]. All three clones no longer produced Hsp70-x with expression of the closely related cytoplasmic Hsp70-1 protein serving as a loading control. Subsequent experiments were performed on at least two of the three clones, and if no difference was observed between clones the combined data are reported.

**Fig 2:**
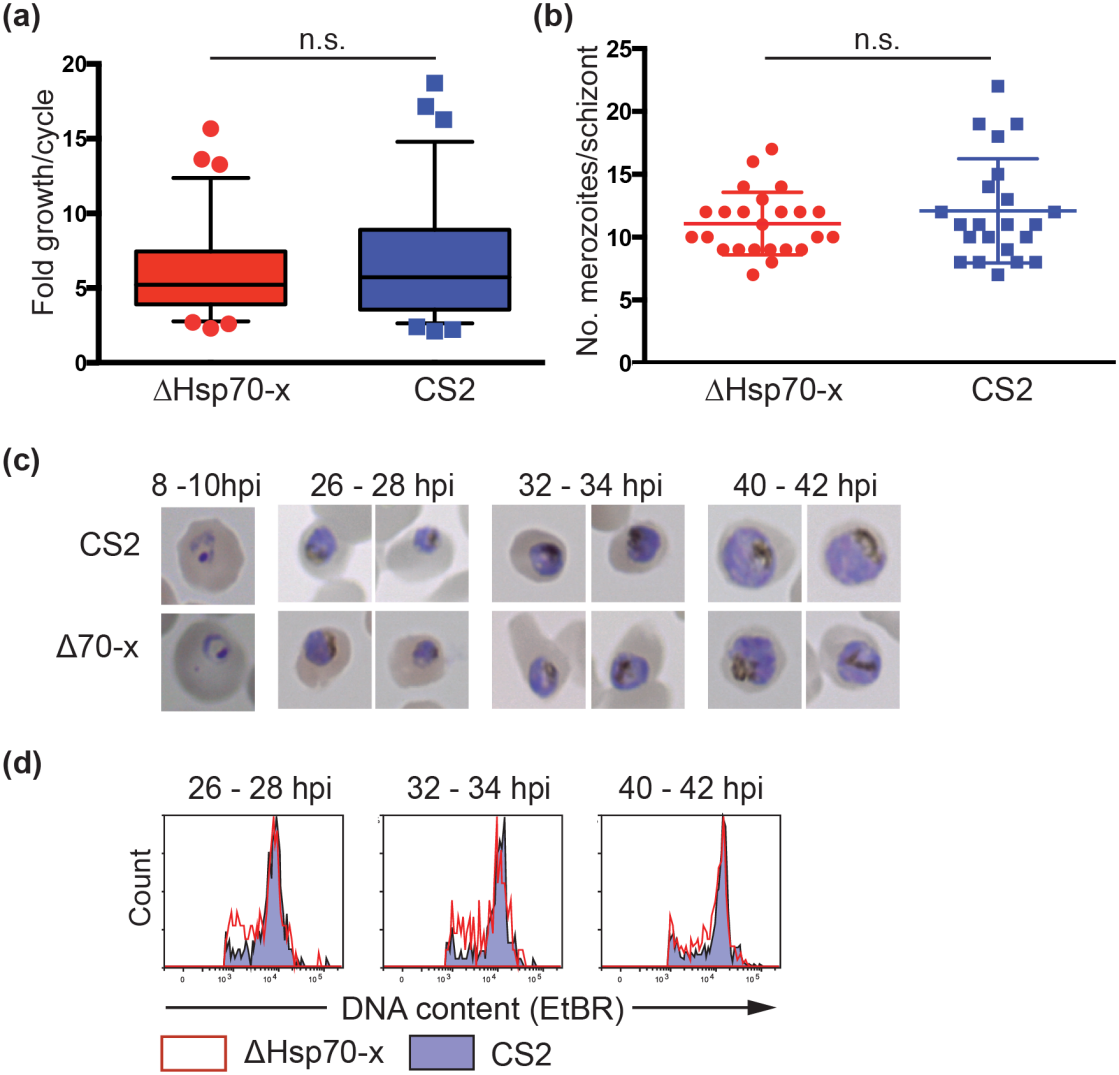
Deletion of *hsp70-x* does not affect parasite growth in the blood stage cell cycle. (a) Measurement of the fold increase cell proliferation per blood stage cycle of ΔHsp70-x and CS2 parasites as assessed by parasite lactate dehydrogenase (LDH) activity indicates the growth of ΔHsp70-x parasites was not significantly (n.s.) slower than CS2. Mann-Whitney p = 0.7, *n* = 36. (b) Light microscopy counts of the mean number of merozoites per schizont in ΔHsp70-x and CS2 parasite lines indicated there was no significant difference. (c) Light microscopy images of four hour synchronized Giemsa stained parasites indicating both lines were morphologically similar. (d) Measurement of the DNA content of ethidium bromide (EtBR) stained ΔHsp70-x and CS2 IE by flow cytometry indicates similar DNA content in the majority of cells at each time point. Parasites were synchronized to a four-hour window and the red trace indicates ΔHsp70-x DNA content compared to CS2 in purple.

The ability to isolate ΔHsp70-x parasites indicated that Hsp70-x was not essential for survival *in vitro*. Growth rate as measured by the fold amplification of the parasites per cell cycle was measured and indicated that the mean fold increase in ΔHsp-70x was not statistically significant to the parental CS2 line (ΔHsp70-x 5.23(3.90–7.46) vs CS2 5.72(3.57–8.91) median (IQR)) (Fig 2a). To further confirm this, the number of merozoites per schizont was counted in each line, as well as the cell cycle length. Merozoite counts revealed no significant difference with each schizont containing a mean of ~11 merozoites (Fig 2b). Microscopy of Giemsa stained IE indicated that ΔHsp70-x appeared similar to CS2 with no obvious abnormalities (Fig 2c). To more accurately quantify changes in maturation through the cell cycle, the DNA content of parasites stained with ethidium bromide was measured by flow cytometry (Fig 2d). This indicated that most parasites had the same DNA content at each timepoint and were maturing at the same rate. There were slightly more younger parasites in ΔHsp70-x than CS2 represented by the ‘tail’ on the left in the 26–28 and 32–34 hpi samples. This is likely indicative of ΔHsp70-x being less well synchronized rather than a slower cell cycle, which would be expected to reduce the overall growth rate and this was not observed.

### Δhsp70-x parasites are equally as susceptible to most stressors as CS2

**Fig 3:**
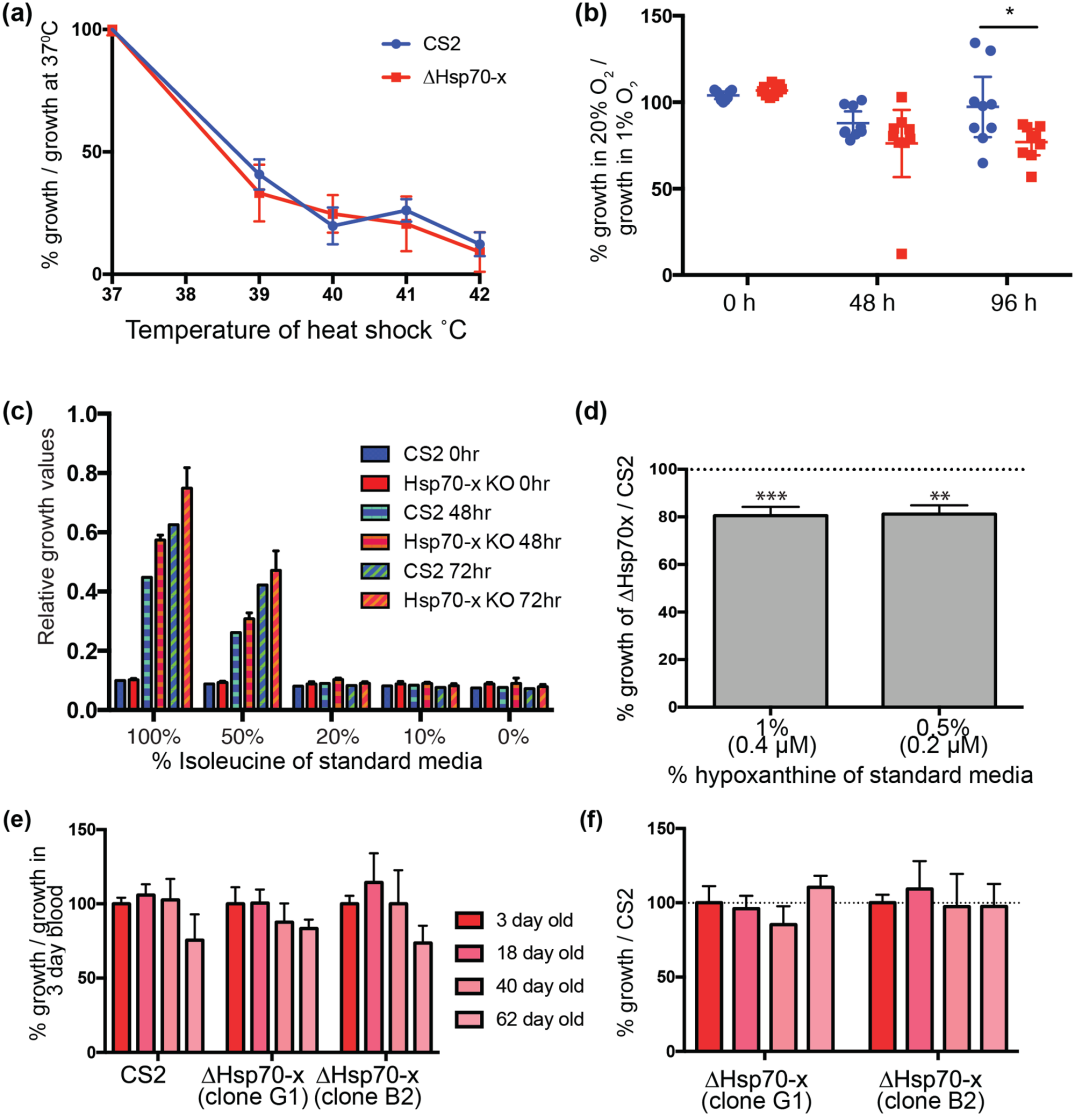
ΔHsp70-x parasites generally resist stress as well as CS2. (a) Growth of ΔHsp70-x and CS2 parasites for 1.5 cell cycles after heat shock treatment of ring stage parasites indicated that growth of the mutant ΔHsp70-x and CS2 were similarly reduced. The ring-stage parasites were heat shocked at indicated temperatures for six hours and the percentage growth relative to parasite growth at 37°C is shown. The parasite lactate dehydrogenase (LDH) assay was used to measured growth and was performed twice in triplicate. (b) Percentage of blood stage parasite growth in 5% CO_2_ in normal air (20% O_2_) as a proxy for oxidative stress, relative to non-stressed cultures in 5% CO_2_, 1% O_2_. Both CS2 and ΔHsp70-x have reduced growth in oxidative stress conditions at 48 h with ΔHsp70-x not recovering as well as some of the CS2 replicates at 96 h. Growth was measured by LDH assay and performed in triplicate, showing the mean with 95% CI * p = 0.02. (c) Growth in media with reduced levels of isoleucine as measured by LDH assay at 0, 48 and 72 h after start of growth period, *n* = 2. For most isoleucine concentrations there was no difference between the ΔHsp70-x and CS2 parasites. (d) With hypoxanthine reduced to 1% (0.4 μM) and 0.5% (0.2 μM) of standard RPMI culture conditions, the growth of ΔHsp70-x was slightly reduced compared to CS2 as indicated by the dashed line at 100% (*n* = 3). Growth was measured by LDH activity after two cycles and was performed with two clones of ΔHsp70-x whose data were combined because they were not significantly different to each other. T-test comparing ΔHsp70-x growth to CS2 p = *** > 0.0001, ** 0.0008. (e) Growth of parasite lines in different aged erythrocytes as measured by LDH activity after 2.5 cell cycles indicates both parasite lines grew less well in older erythrocytes. The erythrocytes were stored at 4°C for the indicated number of days prior to start of the assay. Parasite LDH was measured five days after the start of the assay and is reported as % growth compared to parasites grown in 3 day old blood, *n* = 6. (f) Data from (e) showing the growth of ΔHsp70-x clones compared to control CS2 parasites normalised to 100% for each erythrocyte age group indicates no difference.

Since Hsp70s are important for helping cells cope with heat and other stresses we subjected the ΔHsp70-x parasites to a range of extreme conditions to determine if they were more vulnerable. Ring stage parasites were first heat shocked between 37°C and 42°C for six hours and then allowed to recover for 1.5 cell cycles (~72 h). Lactate dehydrogenase (LDH) activity was measured as a surrogate for parasite growth and indicated that ΔHsp70-x growth was not reduced relative to CS2 (Fig 3a). Next parasite growth in an atmosphere containing standard parasite culture gas (5% CO_2_, 94% N_2_ and 1% O_2)_ or air containing 20% O_2_ and 5% CO_2_ was compared. Parasites prefer a low oxygen environment and LDH assays performed after 48 hours incubation in these atmospheres indicated both parasites grew less well in 20% O_2_ (Fig 3b). After 96 h in 20% O_2_, growth continued to be reduced in both lines but CS2’s growth had become variable between replicates (Fig 3b). It was possible that in some replicates CS2 was able to adapt to high O_2_ conditions, whereas the ΔHsp70-x line was not.

Next, we subjected the parasites to restriction of nutrients normally acquired from human blood plasma via new permeability pathways (NPPs). NPPs are broad specificity anion channels of the erythrocyte membrane permeable to amino acids, purines and vitamins and may be formed by secreted and exported parasite protein as well as co-opting human proteins [13, 44–46]. With Hsp70-x residing in the PV and erythrocyte compartments, this chaperone could be involved in the establishment or efficient functioning of NPPs. We compared the growth of ΔHsp70-x and CS2 parasites in media depleted of two serum nutrients shown to be acquired via NPPs and essential for growth; isoleucine and hypoxanthine [47–49]. Isoleucine restriction was detrimental to both parasite lines, to an equivalent level, with neither line proliferating in 20% or less of standard culture media (Fig 3c). Hypoxanthine restriction to normal plasma levels [50] of 0.4 μM (1% of standard culture media) or 0.2 μM (0.5%) resulted in reduced growth of ΔHsp70-x clones by 20% compared to CS2 (Fig 3d).

Finally we subjected the parasites to stress by culturing them in aged erythrocytes, which have reduced intrinsic chaperone activity, to determine whether host Hsp70s might be compensating for the loss of *P*. *falciparum* Hsp70-x (34). Parasite growth was measured by LDH assay in erythrocytes that had been previously stored at 4°C for 3 to 62 days. Although the growth rates of both lines were reduced in older erythrocytes (Fig 3e), there was no difference between two clones of ΔHsp70-x and CS2 when the erythrocytes were matched for age (Fig 3f). The data indicate that although parasite growth is slightly reduced in aged erythrocytes, loss of Hsp70-x did not put the parasites at a significant growth disadvantage relative to CS2.

### ΔHsp70-x parasites have reduced virulence phenotypes with less efficient PfEMP1 export and cytoadherence

**Fig 4:**
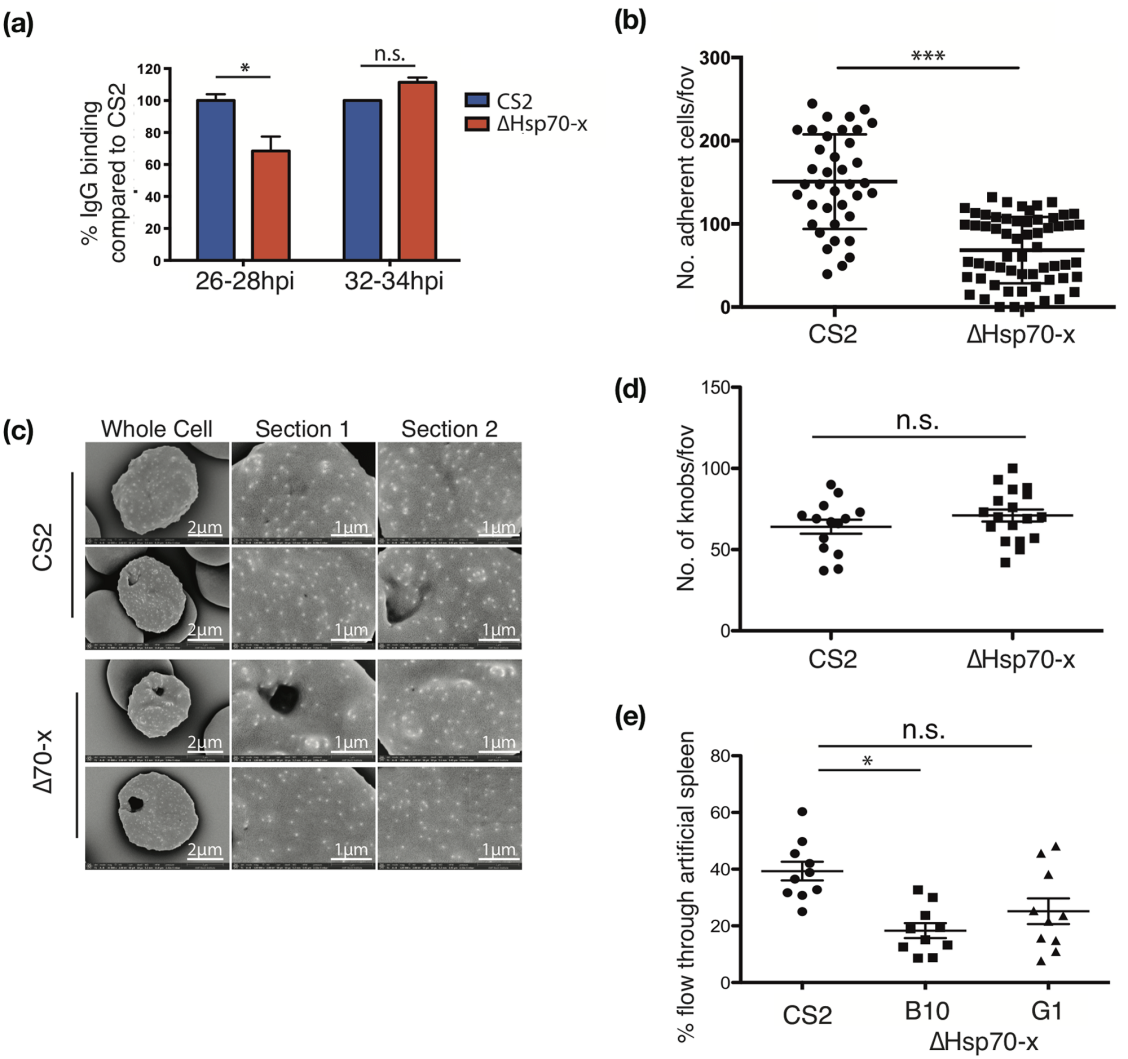
ΔHsp70-x parasites export *Pf*EMP1 less efficiently, bind less well under flow and have altered cell rigidity. (a) Recognition of surface exposed *Pf*EMP1 by human immune sera indicates there is a delay in the deployment of *Pf*EMP1 on the surface of young (26–28 hpi) but not older (32–34 hpi) ΔHsp70-x infected erythrocytes compared to CS2. Flow cytometry was used to measure the mean fluorescence intensity of *Pf*EMP1 on synchronized parasites and was performed in triplicate. T-test *p = 0.04, *n* = 2. (b) Cytoadhesion of infected cells at 32–34 hpi under a 0.05 Pa flow rate to simulate microvasculature conditions indicates that ΔHsp70-x bind significantly less well than CS2. Adhesion was measured as the mean number of cells/view, dot plot shows CS2 geometric mean of 138, ΔHsp70-x geometric mean of 51 in three clones, indicating an overall reduction in binding of 63%. Wilcoxon test *** p < 0.0001 *n* = 3, each performed in triplicate. (c) Representative scanning electron micrographs of CS2 and ΔHsp70-x infected erythrocytes indicate similar morphology and density of knobs. (d) Analysis of number of knobs per field of view of single IE CS2 and Hsp70-x shows no significant difference. (e) Deformability of infected erythrocytes (23–26 hpi) measured by reduced passage through an artificial spleen system of packed micro-beads. The ΔHsp70-x clone B10, is more rigid than CS2 (T-test CS2 v B10 *p = 0.03). Clone G1 also more rigid than CS2 but not significantly so (CS2 v G1 p = 0.11). *n* = 3, each performed in triplicate.

As the only exported chaperone of its class, Hsp70-x may also be necessary for the efficient export and function of *P*. *falciparum* proteins trafficked to and through the erythrocyte cytoplasm. Hsp70-x could be particularly important for the large *Laverania*-specific EMP1 virulence proteins which aid cytoadherence of IE, but are not necessary for *in vitro* culture [2]. *Pf*EMP1 is first exported out of the parasite, across the PVM into the erythrocyte cytosol and then, via membranous lamellae called Maurer’s clefts, is transferred onto knobs at the erythrocyte surface [51]. To determine the efficiency of exposure of *Pf*EMP1 on the IE surface, whole IE were exposed to malaria immune sera from pregnant women [12, 29]. Since the majority of antibodies to the CS2 IE surface are targeted to the VAR2CSA *Pf*EMP1 [30, 52] antibody binding to IE can therefore be used as a surrogate for *Pf*EMP1 exposure on the surface. Antibody binding to the surface of ΔHsp70-x IEs compared to CS2 was reduced by 32% at 26–28 hpi, but became nearly equal at 32–34 hpi. This suggested that *Pf*EMP1 trafficking was slower and less efficient in ΔHsp70-x, but ultimately surface exposed *Pf*EMP1 reached similar levels in ΔHsp70-x as CS2 (Fig 4a).

Efficient cytoadherence to endothelial cells depends on a number of factors, including the surface exposure of *Pf*EMP1 on correctly formed knob structures and the increased rigidity of the IE cytoskeleton [5, 31, 53–55]. The CS2 parasite line in which the ΔHsp70-x clones were derived from stably expresses the single VAR2CSA *Pf*EMP1 allele, which binds to chondroitin sulfate A (CSA) normally present in the placenta [56]. In static conditions ΔHsp70-x and CS2 were equally able to bind to immobilised CSA (Figure A in S1 Fig). However under flow conditions of 0.05 Pa, a similar flow rate as may be experienced in placental microvasculature, ΔHsp70-x binding was reduced by 63% (Fig 4b) [57, 58]. These assays were performed at 32–36 hpi, when *Pf*EMP1 surface expression was equivalent between the knockout and control lines (Fig 4a). At higher flow rates (0.1 Pa) both CS2 and ΔHsp70-x lines were unable to efficiently bind CSA (Figure B S1 Fig). A decrease in cytoadherence of ΔHsp70-x IE under flow could be due to incorrect loading of *Pf*EMP1 into knobs, or the incorrect formation of knob structures to efficiently display *Pf*EMP1. Scanning electron microscopy was performed to examine knob morphology and revealed that both lines produced knobs, and there were no obvious morphological differences in the size and density of knobs between the lines (Fig 4c and 4d).

As knob formation and *Pf*EMP1 surface exposure appears to be similar between CS2 and ΔHsp70-x, yet cytoadherence was reduced, we therefore investigated another factor related to efficient cytoadherence; the rigidity of the IEs. An artificial spleen model mimicking splenic slits measures the ability of the IE to deform as they traverse through a bed of packed 5–25 μM microbeads [32]. ΔHsp70-x IE traversed the bead-bed less efficiently over the measurement period than age-matched CS2 parasites (Fig 4e). At 25–29 hpi 40% of CS2 parasites traversed the spleen, yet for two ΔHsp70-x clones only 18% and 25% traversed, respectively. Older 28–32 hpi parasites from both lines were unable to traverse the bead bed due to increased rigidity (data not shown). The data indicate that the ΔHsp70-x IE become more rigid at an earlier point in the intraerythrocytic developmental cycle than CS2. Collectively, the decrease in rate of surface exposure of *Pf*EMP1 and increased stiffness of the IE membrane in ΔHsp70-x may be the cause of reduced CSA binding under flow.

### Increased expression of exported proteins may be compensating for the loss of Hsp70-x

**Fig 5:**
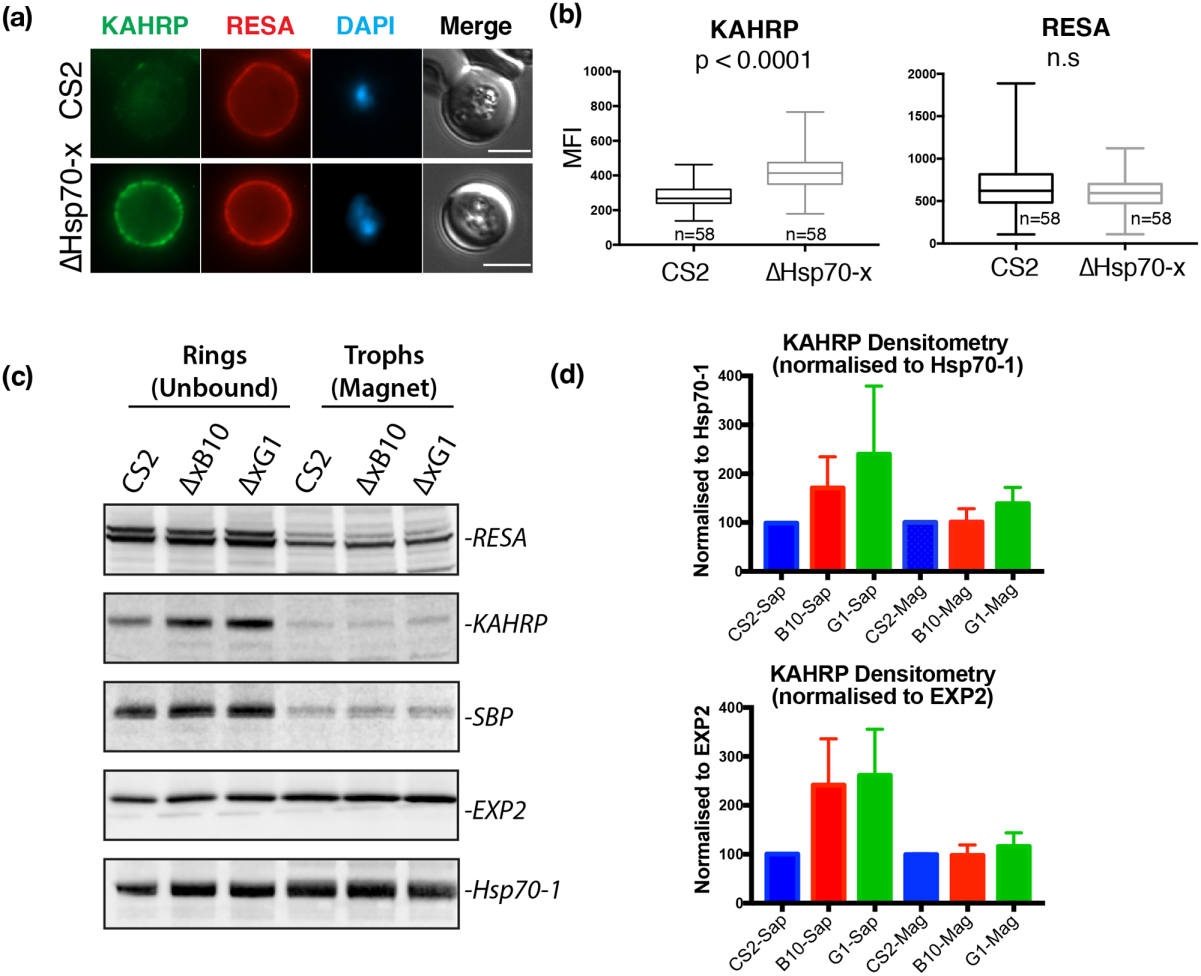
The expression of cytoskeletal binding proteins KAHRP is increased in ΔHsp70-x. (a) Immuno fluorescence microscopy of 16-24hpi parasites indicates expression of the exported, knob localized KAHRP protein but not RESA appears higher in ΔHsp70-x. n = number of cells counted. (b) Mean fluorescence intensity (MFI) measurements of the erythrocyte compartment confirmed the KAHRP signal is higher in ΔHsp70-x compared to CS2 (Mann Whitney test). (c) Western blot analysis of magnet binding (trophozoites) and unbound (ring stage) parasites indicates KAHRP but not RESA or SBP1 appears to be more highly expressed in two ΔHsp70-x clonal lines. Whole purified trophozoites and saponin treated ring stage parasites were analyzed. (d) Densitometry of KAHRP levels from three blots normalized to Hsp70-1 and EXP2 loading controls, indicated that although KAHRP expression was always higher in ΔHsp70-x than CS2 this was not significant (p = 0.1, pairwise comparison, Mann Whitney test).

With the loss of ΔHsp70x expression causing a reduction in cytoadherence and increased stiffness of the IE we examined whether these effects were due to changes in the expression of other exported proteins. Such compensatory changes may have occurred during the generation of the knockout line to counter the loss of Hsp70-x, and uncovering such compensation may help indicate the function of Hsp70-x. As a first method to quickly compare the levels of exported protein in ΔHsp70-x and CS2, immunofluorescence microscopy was performed on highly synchronized parasites. Each line was imaged with the same exposure settings and the mean fluorescence intensity (MFI) of individual cells labeled with antibodies to different exported proteins was measured. Antibodies to the cytoskeletal binding proteins ring exported surface antigen (RESA) and knob associated histidine rich protein (KAHRP) indicated the MFI was significantly higher for KAHRP but not RESA in the mutant (Fig 5a and 5b). Western blot analysis was also performed for KAHRP and RESA as well as exported protein skeleton binding protein 1 (SBP1) which is required for *Pf*EMP1 export, and localises to Maurer’s clefts [59]. As the timing of the expression of these proteins is different, mixed ring and trophozoite stage parasites were used. The culture was magnet purified to separate the rings from the trophozoites which were saponin treated to remove haemoglobin or fractionated as whole cells, respectively. By eye, ring-stage KAHRP appeared to be expressed at slightly higher levels in the two ΔHsp70-x lines (Fig 5c). Densitometry also supported this result however after normalizing for Hsp70-1 and EXP2 the trend was not significant (p = 0.1. Fig 5d). Densitometry of RESA and SBP1 were unchanged in the mutants. The trend towards increased KAHRP expression in ΔHsp70-x IE suggested these and possibly other exported proteins might be up-regulated in ΔHsp70-x parasites.

**Fig 6:**
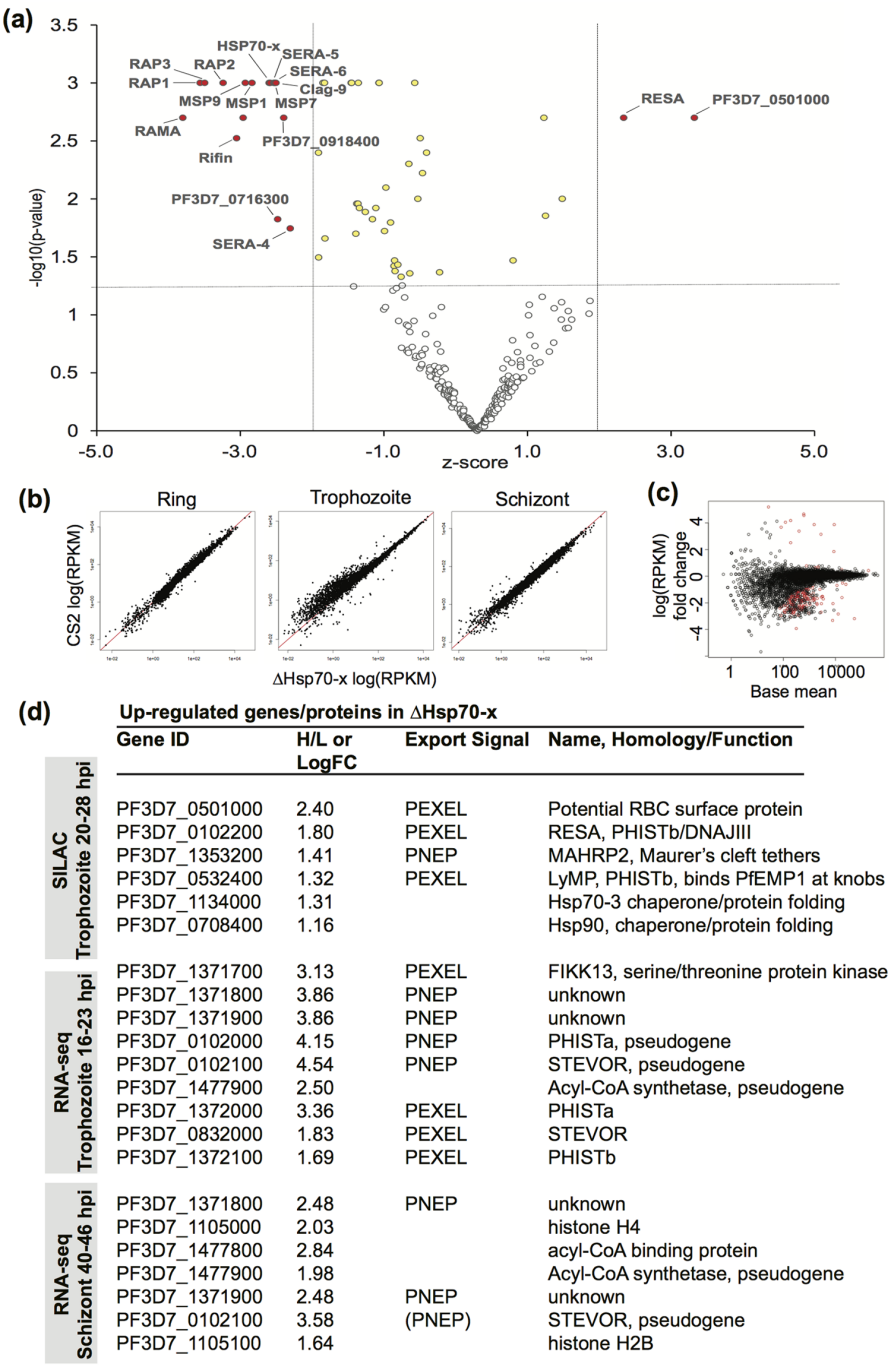
Transcriptional and translational changes in ΔHsp70-x indicate an up-regulation of some exported proteins. (a) Mass spectrometry based SILAC sequencing of proteins indicates several exported proteins are over- and under-expressed in ΔHsp70-x relative to CS2. ΔHsp70-x and CS2 were differentially labeled with heavy (H) and light (L) isotopic forms of isoleucine respectively. Proteins with statistically insignificant Z-score ratios (X-axis) versus—log10 (P-value) (Y-axis) and therefore in ~ 1:1 ratio represent the base of the volcano plot. Proteins at higher levels in ΔHsp70-x only and which satisfy both statistical parameters are labeled in red in the upper right. Those up-regulated proteins that only satisfy one parameter are labeled in yellow. Proteins down-regulated in ΔHsp70-x are similarly coloured and shown in the upper left. (b) Correlation plots of RNA-seq transcripts between wild type and ΔHsp70-x ring, trophozoite and schizont stages show close correlation in most transcripts (close to line x = y). Most differential genes are downregulated in ΔHsp70-x trophozoites and schizonts (points furthest from line). (c) MA plot representing RNA-seq data. The change in expression (log 2 fold change) is plotted against the average of the normalized count values (base mean), of ΔHsp70-x and CS2 transcripts at the trophozoite stage. Red dots indicate differentially expressed genes in ΔHsp70-x. (d) Summary of increased proteins and transcripts by SILAC and RNA-seq. Numbers indicate heavy/light isoleucine ratio (H/L) in SILAC ordered by the ratio, or the log2 fold change in transcripts (LogFC) in RNA-seq data, ordered by significance which takes into account number of transcripts as well. Exported proteins are denoted as PEXEL or no PEXEL (PNEP) or otherwise left blank. Known homology to protein families or potential function identified in final column, including annotated pseudogenes. Pseudogenes may produce a limited numbers of transcripts so small changes can look significant.

For a comprehensive analysis of exported protein levels a quantitative proteomic approach, Stable Isotope Labeling with Amino acids in Cell culture (SILAC), was used to identify up- and down-regulated proteins in ΔHsp70-x parasites compared to CS2. ΔHsp70-x parasite proteins were labeled with heavy (^13^C and ^15^N) isoleucine and CS2 with light isoleucine, and samples were mixed, purified and analysed together. Exported proteins were enriched, by using equinatoxin to perforate only the erythrocyte membrane and to release soluble exported proteins. Lysis and centrifugation also released membranous structures in the host cell, and fragments of erythrocyte membrane and associated proteins. Haemoglobin was removed by passing the samples over nickel resin, which also removed histidine rich proteins. The ratio of Heavy/Light for individual peptides was normalized to the vast majority of proteins that were present at a 1:1 ratio, represented at the base of the ‘volcano plot’ in Fig 6a. A total of 304 proteins were identified and individual proteins identified as up- (upper right) or down- (upper left) regulated in ΔHsp70-x parasites constituted minor subsets (Fig 6a and S1 and S2 Tables). Only two exported proteins RESA (PF3D7_0102200) and a PEXEL-containing uncharacterised protein (PF3D7_0501000), exceeded both P-value and Z-score parameters for up-regulation in ΔHsp70-x (red spots, Fig 6a and 6d and S1 and S2 Tables). Of the four other proteins that exceeded at least one parameter, two were exported (PF3D7_1353200 and PF3D7_0532400) and two were chaperones located within the parasite (yellow spots, Fig 6a and 6d and S1 and S2 Tables). Within the top twenty proteins with H/L ratios >1.25, were also two other chaperones and at least three exported proteins (S1 and S2 Tables).

In contrast to the relatively few proteins over-expressed in ΔHsp70-x, there were many more under-expressed relative to CS2 (Fig 6a, S1 and S2 Tables). There were 16 under-expressed proteins that exceeded both Z-score and P-value parameters and twice as many proteins that exceeded a single parameter (red and yellow spots, respectively, Fig 6a). Hsp70-x peptides were amongst those under-represented in the ΔHsp70-x parasites (Fig 6a, S1 and S2 Tables). In theory there should be no peptides in Hsp70-x parasites but since this protein is very similar to the cytoplasmic chaperone Hsp70-1 [18], a few peptides of the cytoplasmic chaperone may have been counted as being from the exported chaperone. Many of the proteins under-expressed in ΔHsp70-x trophozoites were schizont proteins (eg, rhoptry proteins [RAPs and RhopHs] and merozoite surface proteins [MSPs]).

As both the immunofluorescence and proteomics approaches examined surface and exported proteins in trophozoites, we used RNA-seq to investigate the effects of ΔHsp70-x deletion upon steady-state mRNA levels in tightly synchronized ring, trophozoite and schizont stage parasites. Expression of most genes was closely correlated at each time point, indicating close age-matching of replicates (Pearson correlation coefficient >0.9) (Fig 6b). Each sample also correlated well to the expected time points of the intra-erythrocytic development cycle derived from published microarray data [60] (S2 Fig). The greatest number of differentially expressed genes detected between the two parasite lines occurred at the trophozoite stage. Differences in read counts and fold change are illustrated in Fig 6c; differentially expressed genes are highlighted in red (Fig 6c, S3 Table). Of the small number of up-regulated ΔHsp70-x genes identified in trophozoites and schizonts the majority of these (8/9 at trophozoite, 3/7 at schizont) were predicted to be exported (Fig 6d and S1 and S3 Tables). In comparison, many more genes were identified as down-regulated in ΔHsp70-x trophozoites compared to CS2 (S1 and S3 Tables). The majority of these down-regulated genes encode known schizont stage proteins, involved in processes such as invasion.

## Discussion

Hsp70-x, which localises to the PV and is exported into the host cell via PTEX, is the only known *Plasmodium* exported Hsp70 [18, 61]. Hsp70-x is present only in the *Laverania* sub-genus, which have three major features in common; 1) they infect erythrocytes of all ages, 2) they encode an EMP1 type protein used for cytoadherence in *P*. *falciparum*, and 3) they have large exportomes. We show here that Hsp70-x can be deleted *in vitro*, and the resulting mutant parasites are still able to export *Pf*EMP1, but the process is less efficient and the resulting IE are less cytoadherent and more rigid. Decreased binding in the microvasculature and increased rigidity of these parasites should lead to enhanced splenic clearance, which we recapitulate using an *in vitro* model indicating ΔHsp70-x could be important *in vivo*. The deletion of ΔHsp70-x appears to trigger multiple changes in the transcription and translation of exported proteins in particular, which could in part compensate for the loss of this chaperone. We acknowledge these changes could also be partly a result of the clonal selection process used to isolate the ΔHsp70-x parasites that may have other mutations but the fact that multiple clones performed similarly makes this less likely. The data therefore indicate that although Hsp70-x is not essential for growth it may aid in the efficient export or function of some proteins that are important *in vivo*. To overcome possible adaptive processes using the knock out approach we attempted to rapidly knockdown Hsp70-x by integrating a riboswitch *glmS* into the 3’UTR. Surprisingly there was no significant protein knockdown following glucosamine treatment, and so we have not included these experiments.

*Hsp70-x* is located in the subtelomeric region of chromosome 8, and in a study of Peruvian clinical isolates loss of the adjacent gene histidine-rich protein 2 (*hrp2*) occurred in 40.5% of isolates but only 11.5% lacked *hsp70-x* [62]. This suggests the biological role for Hsp70-x *in vivo* is important as few isolates lose this gene even when neighboring genes are lost. Growth responses to multiple stressors were analysed and identified little change in ΔHsp70-x.

Hsp70-x may play a role in immune evasion, specifically in *Pf*EMP1 export and cytoadherence. Lack of Hsp70-x delayed but did not prevent exposure of *Pf*EMP1 on the surface of the iRBC as measured by reaction to human immune sera and cytoadhesion under static conditions. However, under low-pressure flow conditions reminiscent of flow rates in the placenta, ΔHsp70-x IE were significantly less able to cytoadhere to CSA compared to CS2 at an intra-erythrocytic time point where both lines had equal levels of surface-exposed *Pf*EMP1. Neither parasite line was able to efficiently cytoadhere under high flow conditions, indicative of the reduced cytoadhesive properties of placental binding parasites such as CS2 in general [63]. We do not believe reduced cytoadherence is due to a sub-population of slower developing parasites in ΔHsp70-x as we performed the experiments when equal amounts of surface exposed *Pf*EMP1 are present, and we see no evidence of two populations one more delayed than the other, nor in the cytoadherence and cellular rigidity. Slower delivery of *Pf*EMP1 to the surface may be due to reduced export efficiency although a less synchronous ΔHsp70-x population could also be contributing (see below). Slower *Pf*EMP1 export may also be due to disruption of J-dots, chaperone dense regions that contain Hsp70-x and colocalise with *Pf*EMP1 during its export to the cell surface [18] which warrants further study.

Cytoadherence can be affected by knob formation and/or cytoskeletal changes in the erythrocyte induced by parasite derived proteins. Although ΔHsp70-x IE parasites appeared to have normal knob density, they were retained more efficiently in an artificial spleen model, indicating increased rigidity. In the presence of functioning *Pf*EMP1 increased stiffness leads to increased cytoadhesion [64], however in the absence of functional surface *Pf*EMP1 increased stiffness would further reduce cytoadherence and lead to enhanced clearance by the immune system *in vivo*. Deletion of some exported proteins is known to decrease the rigidity of the RBC membrane [5], and therefore increased expression of these proteins might be expected to escalate membrane rigidity. This was supported by microscopy of the spectrin-binding KAHRP in ΔHsp70-x parasites which indicated it was over-expressed relative to CS2, and thus may be contributing to increased rigidity. We note however that although not specifically measured, the knobs of ΔHsp70-x appeared normal despite higher KAHRP expression.

To obtain a broader picture of altered protein expression in ΔHsp70-x IE, mass spectrometry based protein quantification of exported proteins was performed. The data was enriched for exported proteins by erythrocyte membrane lysis with equinatoxin, which also identified cytoskeleton binding proteins. This is probably due to small light weight membrane fragments remaining in the supernatant. Quantitative SILAC analysis identified RESA and a protein of unknown function with high confidence, although analysis was only performed once. The increase in RESA levels by SILAC was not supported by microscopy and western blots. RESA’s increase by SILAC could be due to increased solubility and release from the cytoskeleton in ΔHsp70-x trophozoites. PF3D7_0501000 is predicted to be exported and may be on the iRBC surface [65] although we do not have antibodies available to confirm elevated levels in ΔHsp70-x. KAHRP, being rich in histidine residues, was probably lost during the nickel bead step used to remove hemoglobin and was not detected. Other over-expressed proteins were MAHRP2 (PF3D7_1353200) and LyMP, (PF3D7_0532400) which have been shown to respectively help tether Maurer’s clefts to the erythrocyte surface, and the ATS of certain *Pf*EMP1 to the cytoskeleton [66–68]. Over-expression of these cytoskeleton binders could also have contributed to the increased rigidity [64].

Since the proteomics approach was enriched for exported trophozoite proteins we employed RNA-seq to investigate genome-wide changes in the major asexual blood developmental stages of the parasite; rings, trophozoites and schizonts. Relative to CS2, genes up-regulated in ΔHsp70-x encode several exported proteins in both the trophozoite and schizont stages. Like LyMP, many of the genes with up-regulated transcripts encode exported proteins with PHIST (*Plasmodium* helical interspersed sub-telomeric) domains; these proteins may bind and modify the erythrocyte’s cytoskeleton [68]. The most up-regulated gene in ΔHsp70-x trophozoites was the exported kinase FIKK13 [69, 70]. The function of FIKK13 is unknown but it may have a role in cytoadherence and/or membrane rigidity as knockouts of the exported FIKK4.2 and FIKK12 kinases affect these phenomena [71, 72]. We note with RESA, that an increase in transcript levels did not produce a rise in protein abundance by microscopy and western blot and so whether or not a gene upregulated by RNA-seq leads to increased protein levels will need to be validated for each gene.

Although we carefully attempted to age match ΔHsp70-x and CS2 trophzoites and schizonts there were a number of transcripts and proteins identified as down expressed by RNA-seq and SILAC. These tended to be for late stage invasion proteins and it is possible that ΔHsp70-x parasites were slightly less synchronous than CS2 meaning the proportion of older parasites was a little lower in ΔHsp70-x. The mutant may have been more difficult to synchronize by sorbitol because their NPPs were less active. The fact that ΔHsp70-x was slightly more sensitive to restriction of imported hypoxanthine suggests further evaluation of NPP function is warranted.

Hsp70-x would be expected to have a diverse set of binding partners due to its dual location in the PV and in the host cytosol [16, 18]. To further understand Hsp70-x’s function we attempted to directly identify its client proteins by immunoprecipitation with IgG raised to the less conserved peptide binding and ‘lid’ regions of Hsp70-x to prevent cross reactivity with other parasite Hsp70s. This approach however failed to identify strong binding partners possibly because the IgG was competing for the same binding site as the chaperone’s client proteins. Immunoprecipitation of the PTEX subunits PTEX150, EXP2 and Hsp101 does co-purify Hsp70-x suggesting it may be assisting Hsp101 to unfold cargo or might be cargo itself [12, 17, 61]. In silico modeling has suggested that Hsp70-x may bind a range of Hsp40s [73], and chaperone rich J dots containing both Hsp70-x and two Hsp40s have been identified [18]. It has recently also been suggested that host Hsp70s can bind to parasite derived Hsp40s [74] supporting the idea that Hsp70-x is not the only Hsp70 co-opted by the parasite for functions in the host cell, however it is the only parasite derived Hsp70 present in the host cell.

## Conclusions

We show that Hsp70-x is not necessary for survival of *P*. *falciparum* in mature erythrocytes and is not essential for export of *Pf*EMP1. Our data support the hypothesis that Hsp70-x assists efficient export and functioning of some exported proteins that contribute towards virulence. Δ*hsp70-x* parasites have undergone a large number of gene regulatory changes that may be compensatory, and have resulted in mild but significant phenotypes such as slower export of *Pf*EMP1, reduced cytoadherence and increased erythrocyte rigidity. By enhancing large-scale protein export Hsp70-x may contribute to the virulence of *Laverania* parasites in their primate hosts.

## Supporting information

S1 Fig Cytoadhesion in static and flow conditions. (a) Static adhesion of IE to CSA bound to plastic dish. Measured by light microscopy of number of IE/mm^2^, n = 3, no statistical difference between lines. (b) Cytoadhesion of infected cells at 32–34 hpi under a 0.05 Pa and 0.1 Pa flow rate. Adhesion was measured as the mean number of cells/view, box interquartile range and whiskers min-max, *n* = 3, each performed in triplicate.
(PDF)

S2 Fig RNA-seq correlation with microarray data indicating close age matching. Correlation plot of normalized expression values (RPKM) against microarray data [60] shows good correlation between biological replicates and between wild type and ΔHsp70-x. Schizont samples have some correlation with ring stage microarray transcripts as expected. WT indicates CS2 and KO indicates ΔHsp70-x, two biological replicates performed at each time point (rings, trophozoites and schizonts). V indicates time points taken post invasion in [60].
(PDF)

S1 Table List of all differentially expressed proteins and transcripts identified by SILAC and RNA-seq. Homology, function and export of proteins and genes identified are those corresponding to PlasmoDB Release 29. Proteins are identified being EXPORTED if they contain a PEXEL sequence, or PNEP if known to be exported but lacking a PEXEL sequence. SILAC proteins were designated up-regulated if H/L ratio ≥ 1.25 and down-regulated if ≤ 0.75. Proteins are color coded as per Fig 6 and the number of H and L peptide pair observations are indicated. For RNA-seq, maximal expression at stage sampled determined to be “yes” transcription was 24–36 hpi for trophozoites or 36–0 hpi for schizonts as determined from data available from PlasmoDB. RNA–seq downregulated trophozoite proteins only showing top hits, full data in S3 Table.
(XLSX)

S2 Table Total proteomics data for SILAC analysis. (XLSX)

S3 Table Total RNA-seq data for all genes in each biological replicate. CS2 (WT) and ΔHsp70-x (KO) synchronised with sorbitol to within 8 hours window and grown separately for at least three cycles to get biological replicates 1 and 2 at three life stages; rings, trophozoites (trophs) and schizonts (schiz). Tab1 indicates RPKM, Tab2 indicates Degust differential expression analysis of WT and KO trophozoite stage shown with false discovery rate (FDR).
(XLSX)
