# Supplementary figures and images for: The exported chaperone Hsp70-x supports virulence functions for Plasmodium falciparum blood stage parasites
Source: PLoS One. 2017 Jul 21;12(7):e0181656. doi: 10.1371/journal.pone.0181656 (PMC5521827; doi:10.1371/journal.pone.0181656)

Fig. S1

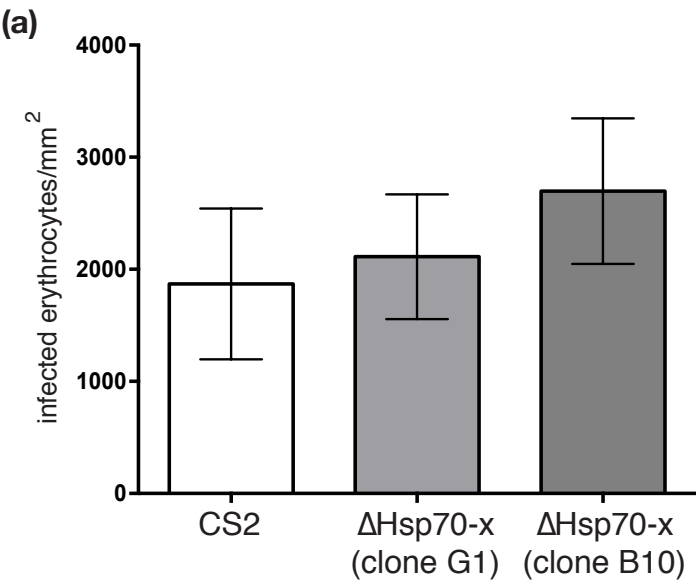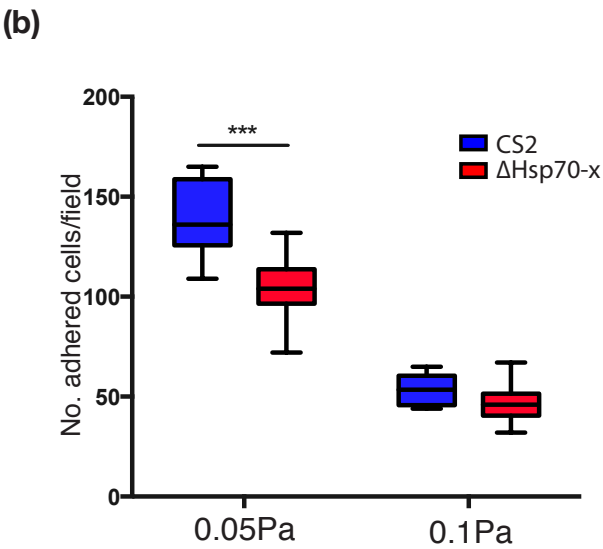

Supplement: S1 Fig — (a) Static adhesion of IE to CSA bound to plastic dish. Measured by light microscopy of number of IE/mm2, n = 3, no statistical difference between lines. (b) Cytoadhesion of infected cells at 32–34 hpi under a 0.05 Pa and 0.1 Pa flow rate. Adhesion was measured as the mean number of cells/view, box interquartile range and whiskers min-max, n = 3, each performed in triplicate. (PDF) [file pone.0181656.s001.pdf]

Fig. S2

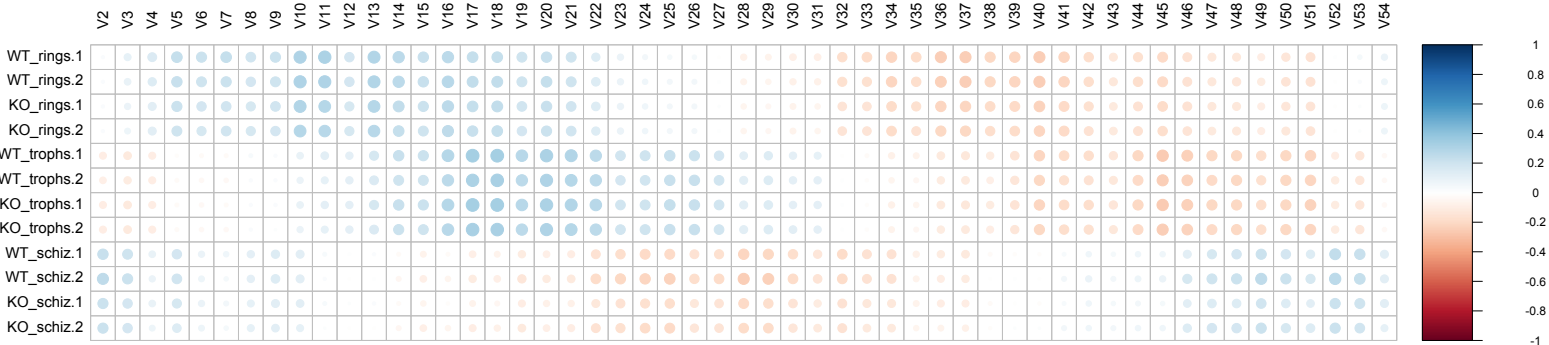

Supplement: S2 Fig — Correlation plot of normalized expression values (RPKM) against microarray data [60] shows good correlation between biological replicates and between wild type and ΔHsp70-x. Schizont samples have some correlation with ring stage microarray transcripts as expected. WT indicates CS2 and KO indicates ΔHsp70-x, two biological replicates performed at each time point (rings, trophozoites and schizonts). V indicates time points taken post invasion in [60]. (PDF) [file pone.0181656.s002.pdf]
